# The IL6-like Cytokine Family: Role and Biomarker Potential in Breast Cancer

**DOI:** 10.3390/jpm11111073

**Published:** 2021-10-24

**Authors:** Carlos Martínez-Pérez, Charlene Kay, James Meehan, Mark Gray, J. Michael Dixon, Arran K. Turnbull

**Affiliations:** 1 Breast Cancer Now Edinburgh Research Team, MRC Institute of Genetics and Cancer, Western General Hospital, University of Edinburgh, Edinburgh EH4 2XU, UK; charlene.kay@ed.ac.uk (C.K.); mike.dixon@ed.ac.uk (J.M.D.); arran.turnbull@ed.ac.uk (A.K.T.); 2 Translational Oncology Research Group, MRC Institute of Genetics and Cancer, Western General Hospital, University of Edinburgh, Edinburgh EH8 9YL, UK; james.meehan@ed.ac.uk (J.M.); mark.gray@ed.ac.uk (M.G.)

**Keywords:** breast cancer, cytokine signalling, IL6ST, gp130, biomarkers, translational research

## Abstract

IL6-like cytokines are a family of regulators with a complex, pleiotropic role in both the healthy organism, where they regulate immunity and homeostasis, and in different diseases, including cancer. Here we summarise how these cytokines exert their effect through the shared signal transducer IL6ST (gp130) and we review the extensive evidence on the role that different members of this family play in breast cancer. Additionally, we discuss how the different cytokines, their related receptors and downstream effectors, as well as specific polymorphisms in these molecules, can serve as predictive or prognostic biomarkers with the potential for clinical application in breast cancer. Lastly, we also discuss how our increasing understanding of this complex signalling axis presents promising opportunities for the development or repurposing of therapeutic strategies against cancer and, specifically, breast neoplasms.

## 1. Introduction

Breast cancer (BC) is a heterogeneous disease comprising well-characterised molecular subtypes that differ in their underlying biology, response to treatments, and prognosis. As with all cancer types, biomarkers with prognostic and/or predictive power are essential tools in the clinical management of this disease, with the oestrogen receptor ⍺ (ER) and the human epidermal growth factor 2 receptor (HER2) being the foremost biomarkers in BC. Assessment of both receptors to help select patients likely to respond to endocrine and HER2-targeted therapies has been established in clinical practice for many decades and has considerably improved the prognosis and survival for patients with hormone-dependent and HER2-overexpressing BC [1,2]. 

Despite said advances, many challenges remain in the management of BC, particularly as it pertains to advanced disease. In order to meet these needs, extensive research efforts are devoted to gaining a better understanding of the underlying complexity of the disease, as well as to identifying and validating potential molecular markers that might enable better patient stratification and treatment selection [2,3,4]. Valuable markers are typically involved in and serve as surrogates for cancer-promoting mechanisms or biological processes known to be altered by disease. Discovery studies continue to identify novel biomarkers predictive of BC development and progression, which can be differentially-expressed or mutated proteins or genes, as well as other genomic markers such as microRNAs or long non-coding RNAs [5,6,7,8].

Here, we review the role of IL6-like cytokines in BC and summarise evidence on the role of members of this ligand family and their receptors as biomarkers, both based on their expression levels or the presence of polymorphisms. Recent years have seen a wealth of evidence reported on this, given the central role of this signalling axis in many cancer-related processes. To our knowledge, this is the most comprehensive review to date on the role and biomarker potential of this cytokine family in breast neoplasms.

## 2. The IL6-like Cytokine Family

Cytokines are a superfamily of small polypeptide regulators involved in cell signalling and the regulation of health and disease. They are often subdivided into families according to their features [9,10,11]. As interleukin-6 (IL6) is the best characterised cytokine of its kind, the group of cytokines with similar structural features and signalling machinery is referred to as the IL6 or IL6-like family. This is also referred to as the gp130 family, as the central feature of this group of cytokines is the transmembrane signalling receptor glycoprotein 130, one or more molecules of which are found in all oligomeric signalling complexes. This signal transducer is also known as CD130, IL-6 receptor subunit β (IL6Rβ) or IL6 signal transducer (IL6ST, which is also its gene name). 

Besides the eponymous IL6, other canonical members of this cytokine family are interleukin-11 (IL11), ciliary neurotrophic factor (CNTF), leukemia inhibitory factor (LIF), oncostatin M (OSM), cardiotrophin 1 (CT1), cardiotrophin-like cytokine (CLC) and neuropoietin (NPN). Interleukin-31 (IL31) is often described as a member of this family, although its signalling complex does not include gp130/IL6ST, but other related signalling and non-signalling receptors [12,13,14]. Other cytokines, such as interleukin-27 (IL27), interleukin-35 (IL35) and interleukin-37 (IL37), have been described by different authors as belonging to either the IL6 or IL12 cytokine families [15,16,17,18,19,20]. Indeed, phylogenetic analysis has shown a close relationship between both groups [21,22,23]. 

Cytokines act as extracellular ligands, binding transmembrane receptors with high affinity to form oligomeric protein complexes. These lead to the formation of gp130/IL6ST homo- or heterodimers (depending on the cytokine and its respective receptors), which trigger intracellular signalling (see graphical abstract). The diversity of ligand-receptor complexes that can be formed, together with signalling through a shared, ubiquitously expressed transducer [24] and interaction with varied downstream regulators, make this cytokine group a highly pleiotropic protein family, involved in a wide range of biological functions, both in vitro and in vivo [25].

IL6-like cytokines exhibit a long chain ‘four-helix bundle’ topology. This consists of four tightly packed ⍺ helices of 15–22 residues in length arranged in two pairs of anti-parallel helices connected by three polypeptide loops [9,25]. Each ligand then associates with a specific set of receptors which can be classified as non-signalling or signalling. ‘Non-signalling’ receptors (also known as ⍺ receptors) are only required by some ligands (namely IL6, IL11, CNTF and CLC) and are involved in the formation of the signalling complex, but do not actively participate in intracellular signalling; their cytoplasmic regions determine intracellular distribution in polarised cells but lack signalling capacities [26]. ‘Signalling’ receptors (also known as β receptors) are required by all ligands, as they are transmembrane proteins whose cytoplasmic domains activate the signalling machinery; gp130/IL6ST is the signalling receptor common to all family members. Where both kinds of receptors are required, the association between the ligand and the non-signalling receptor is typically the limiting step for complex formation and the subsequent activation of downstream signalling, as the ligand can bind a non-signalling receptor with high affinity on its own, but only binds the signalling receptor when in the presence of said non-signalling receptor [27]. 

**Figure 1:**
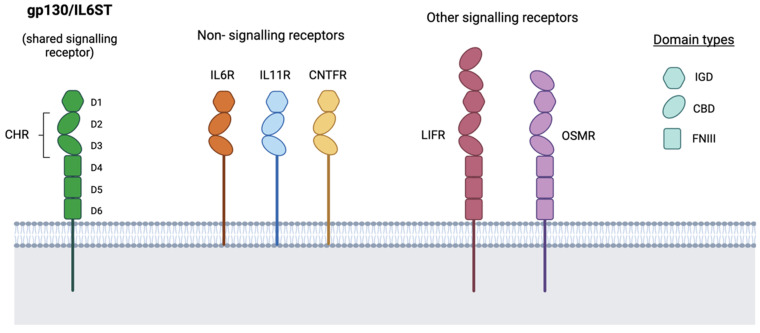
Structure of receptors in the IL6-like family. Receptors for cytokines in the IL6-like family present a modular structure with conserved motifs. Both signalling and non-signalling receptors include a single immunoglobin-like domain (IGD) and a cytokine-homology region (CHR), made up of cytokine-binding domains (CBD). Signalling receptors also present a membrane-proximal element including several copies of a fibronectin type III-like (FNIII) domain. The ectodomain of the shared signal transducer gp130/IL6ST consists of 6 domains, with the 3 membrane-distal ones (D1-D3) being essential for binding to the cytokine (and the non-signalling receptor, where this is required). Other signalling receptors, such as LIFR and OSMR, present larger ectodomains consisting of variations of this modular structures.

The receptors in this family are modular in form and present distinct structural motifs in their extracellular region (or ectodomain): a single immunoglobulin-like domain, a cytokine homology region and, in signalling receptors, a third element including several copies of the fibronectin type III-like domain [9] (see Figure 1 and next section). While all family members bind gp130/IL6ST, their differential affinity for other receptors to form their respective complexes is central to the complex specificity of signalling through this cytokine family. The receptors associated with each member of the cytokine family are summarised in Table 1. Cytokines in the IL6 family are characterised by the existence of 3 topologically discrete sites (I, II and III) that act as functional epitopes for interaction with their receptors. Mutagenesis studies have shown that the specificity of these sites is dictated by a small number of residues in close spatial proximity, some of which are conserved across members of the family [9,25]. 

Site I, used only by some cytokines, is a binding site for non-signalling receptors only (e.g., IL6R for IL6 or IL11R for IL11). A recent study has reported on the different mechanisms for complex formation, evidencing the biological specificity for each ligand-receptor pair [28]. Consistently across all family members, site II is always the binding site for the shared receptor gp130/IL6ST. Site III is always used for association with a second signalling receptor, such as gp130/IL6ST, LIFR or OSMR, depending on the ligand. IL6 and IL11 have been shown to use sites II and III to bind different regions of the same gp130/IL6ST molecule [9]. Following this receptor recognition, higher order complexes are formed combining 2 ligands and their respective receptors, as described in the following section. 

## 3. Soluble Receptors and Signalling Modes

**Figure 2:**
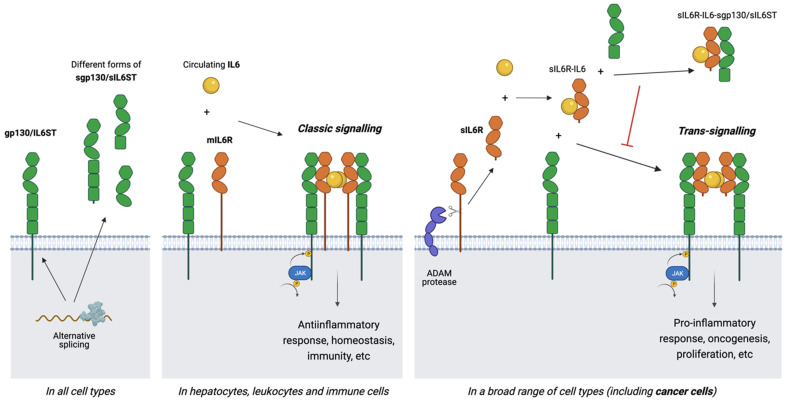
Signalling modes in IL6-like cytokine signalling. The shared signalling receptor gp130/IL6ST is ubiquitously expressed across all cell types in its full-length, membrane-bound form. Different soluble forms (sgp130/sIL6ST) are also produced, mainly through alternative splicing. Different cell types produce membrane-bound or soluble forms of non-signalling receptors such as IL6R (mIL6R or sIL6R, respectively). Receptor availability will determine what signalling mode is induced by a cytokine. In the classic signalling mode, IL6 forms a hexameric signalling complex by binding mIL6R and gp130/IL6ST. Alternatively, circulating sIL6R can act a cytokine agonist, capturing IL6 to trigger trans-signalling, associated with pro-inflammatory and pro-carcinogenic responses. In turn, sgp130/sIL6ST can act as a cytokine antagonist, sequestering the sIL6R-IL6 complex and inhibiting trans-signalling.

As the prototypical and best-characterised member of its cytokine family, IL6 represents the best model to describe the complex signalling machinery observed in this family. Importantly for the scope of this review, IL6 also plays an important role in BC, so the description of its specific signalling partners and modes will be informative to the sections focusing on this disease. IL6 binds the non-signalling receptor IL6R at the cytokine’s site I before the IL6-IL6R complex can bind the signalling receptor gp130/IL6ST using IL6′s sites II and III. Two such complexes then dimerise to form a final ternary complex with a hexameric conformation and stoichiometry that includes two molecules each of IL6, IL6R and gp130/IL6ST [29,30,31] (see Figure 2). It is this complex that creates the gp130/IL6ST homodimer necessary to activate downstream signalling in the cytoplasm. While a tetrameric complex model (comprising one molecule each of IL6 and IL6R and two molecules of gp130/IL6ST) has also been proposed [32], the higher order hexameric conformation, similarly described in other IL6-like cytokines such as IL11 [33] and CNTF [34], has become the canonical model for complex formation and signal activation [35]. 

Two different modes of IL6 signalling have been described that are determined by the existence of two different forms of the IL6 receptor ⍺ (see Figure 2): classic IL6 signalling involves the full-length, membrane-bound form (mIL6R), while trans-signalling involves the soluble form (sIL6R), produced from mIL6R, mainly by ectodomain cleavage or shedding by a disintegrin and metalloproteinase domain-containing protein (ADAM10 and ADAM17) [36,37] or, in a smaller proportion, by alternative splicing [38,39]. Recently, a third signalling mode referred to as trans-presentation has been described, by which IL6 binds mIL6R on the surface of a dendritic cell and the resulting complex is then presented to adjacent CD4+ T cells, leading to h17 cell differentiation [40]. However, this mechanism has not yet been observed in human models.

As gp130/IL6ST is ubiquitously expressed in all cell types [24], the form of IL6R available will determine whether IL6 elicits signalling through the classic or trans-signalling routes which, importantly, have been shown to have divergent functions (see Figure 2). Classic signalling is limited to mIL6R-expressing hepatocytes, leukocytes, and immune cells, and has been shown to control homeostasis and promote anti-inflammatory responses [41,42]. In contrast, evidence has shown that sIL6R is produced by a broad range of cell types, including malignant types such as BC cells, which produce sIL6R endogenously. sIL6R can circulate through the bloodstream where it binds up to 70% of the circulating IL6, thus increasing the cytokine’s half-life and bioavailability and acting as a carrier for its delivery to gp130/IL6ST, available in the membrane of all cell types [43,44]. In this way, trans-signalling broadens the target cell repertoire of IL6, enabling response to the cytokine in cells lacking mIL6R. Depending on the levels of sIL6R produced, trans-signalling can take place as a paracrine action or at both local and systemic levels [45]. Trans-signalling has been linked to pro-inflammatory effects and the observed role of IL6 in chronic diseases and cancer [27,42,46,47]. Trans-signalling mechanisms have also been described for the IL6-like cytokines IL11 and CNTF through soluble forms of their respective non-signalling receptors, sIL11R and sCNTFR [48,49]. The soluble receptors sIL6R, sIL11R, and sCNFTR are considered agonists, since they act as ligand-binding receptors that enable cytokine presentation and complex formation [47]. 

The other essential receptor in all IL6 signalling is, obviously, gp130/IL6ST. While this is ubiquitously expressed, its role is also complicated by the existence of circulating forms. The extracellular portion of gp130/IL6ST consists of 6 domains (see Figure 1): 1 N-terminal immunoglobulin-like domain (IGD), 2 cytokine-binding domains (CBD) and 3 fibronectin type III-like (FNIII) domains. The 3 membrane-distal domains are essential for ligand recognition, since the 2 CBDs (D2-D3) form the cytokine homology region (CHR) and the IGD (D1) is also required for the receptor to be functionally responsive to the cytokine; the 3 membrane-proximal FNIII domains (D4-D6) provide the right spatial orientation to enable formation of the hexameric receptor complex and signal transduction [35,50,51]. At least 4 soluble forms of gp130/IL6ST (sgp130/sIL6ST) have been reported, which consist of the entire (D1-D6) or part (D1-D3 or D1-D4) of the ectodomain [39,42,52], often presenting stabilising glycosylations [53]. These soluble receptors, found at levels of up to 400ng/mL in the blood [54,55,56], are produced mainly through alternative splicing, although ectodomain shedding might also contribute to a very small proportion of their production [39,57,58]. 

Unlike soluble non-signalling receptors like sIL6R, sgp130/sIL6ST acts as a cytokine antagonist, competing with membrane-bound gp130/IL6ST to bind the circulating IL6-sIL6R complex and, thus, selectively blocking IL6 trans-signalling [59,60,61] (see Figure 2). All identified forms of sgp130/sIL6ST include the N-terminal cytokine-binding portion of the receptor. To date, there is no clear evidence of differential antagonistic abilities between the different known forms of sgp130/sIL6ST [42,62]. Evidence has shown that sgp130/sIL6ST can also inhibit IL11 trans-signalling [48,63]. Soluble forms of the signalling receptors OSMR and LIFR have also been reported [64,65], which act as antagonists for OSM and LIF signalling, respectively. 

Given the opposing effects of sIL6R and sgp130/sIL6ST on IL6 signalling, and the fact that their plasma levels remain relatively stable (40–75 ng/mL for sIL6R [66] and 250–400 ng/mL for sgp130/sIL6ST [56,67]), these soluble forms of the receptors act as a buffer for circulating IL6. Plasma levels of this cytokine vary broadly by up to six orders of magnitude between health and disease and in response to different local and systemic processes [39]. Thus, this buffering mechanism might prevent unspecific overstimulation by IL6 trans-signalling unless systemic or local IL6 levels surpass a certain threshold. Research has also reported cell type-specific expression patterns for the different existing forms of sgp130/sIL6ST, which might enable local fine-tuning of the antagonistic effect on IL6 trans-signalling [57].

## 4. Shared Cytokine Signalling: Pleiotropy, Redundancy and Specificity

Cytokine-driven dimerisation of gp130/IL6ST leads to signal transduction and activation of 3 major downstream pathways: the Janus-activated kinase—signal transducer and activator of transcription (JAK/STAT) pathway, the Ras-Raf mitogen-activated protein kinase (MAPK/MERK/ERK) signalling cascade, and the phosphoinositol-3 kinase—protein kinase B/Akt (PI3K/AKT) pathway. This versatile signalling cascade is initiated by tyrosine kinases in the JAK family, such as JAK1, JAK2 and TYK2, which can be found constitutively associated with the cytoplasmic region of gp130/IL6ST by a non-covalent bond. Dimerisation of gp130/IL6ST causes auto-phosphorylation and activation of JAK. One cascade can see JAK phosphorylating the signal transducer and activator of transcription 3 (STAT3), leading to its dimerisation and translocation to the nucleus, where it modulates proliferation and cell survival. JAK can also activate the SH2 domain-containing cytoplasmic protein tyrosine phosphatase (SHP2), which in turn activates the Ras/Raf pathway, leading to the hyperphosphorylation of mitogen activated protein kinases (MAPK) and triggering its increased serine/threonine kinase activity and complex downstream cascade, which includes various transcription factors linked to cell growth [68,69]. Thirdly, JAK can also activate the PI3K/AKT pathway. These signalling pathways are under regulation by a number of negative-feedback mechanisms, including temporal attenuation of the activity of SHP2 and the induction of the suppressor of cytokine signalling (SOCS) protein family [70]. 

These three main signalling pathways, with their own complex and pleiotropic effects, lead to the wide range of functions of IL6 and related cytokines in the healthy organism and in diseases, such as immune disorders and cancer. The tumour-promoting effects of these cytokines include both cancer cell-intrinsic processes, such as cell proliferation, differentiation, survival, invasion and metastasis, and extrinsic processes that affect the tumour microenvironment (TME), such as modulation of inflammation and angiogenesis [71,72]. Reliance on gp130/IL6ST as a shared signal transducer enables a certain level of functional redundancy across family members [68]. Despite relative selectivity in ligand-receptor recognition, structural similarities still allow for some level of receptor promiscuity, which can lead to crosstalk, where a cytokine associates with receptors other than their own with lower affinity. In vitro studies have previously reported non-canonical cytokine-receptor complexes such as OSM-LIFR [12] or CNTF-IL6R [73], which might widen a cytokine’s target spectrum, enabling them to elicit effects normally associated with other ligands in the family.

Nevertheless, there is extensive evidence of significant functional specificity for different cytokines in vivo, with specific members exerting unique functions or the same cytokine being able to elicit different responses in different cell types [17]. How exactly this cytokine family circumvents its built-in redundancy to achieve specificity remains unclear, although a number of features in this family’s complex signalling machinery are likely to contribute to this modulation. The expression patterns for the different cytokines are different across cell populations and can be modulated by the extracellular matrix, while levels or bioavailability of circulating cytokines might also vary. The same is true of the expression levels of different receptors which, as previously mentioned, are often the limiting factor in cytokine signalling and can determine the signalling mode triggered. 

The complex formation process relies on a sophisticated network of interactions between each cytokine and the relevant non-signalling and signalling receptors. The complex extracellular portion of gp130/IL6ST enables additional functional complexity, as multiple domains and regions are involved in ligand recognition and activation [35]. This explains how, for instance, IL6 is only able to associate with gp130/IL6ST as part of a binary IL6-IL6R complex. Research has shown that different cytokines bind different specific residues in gp130/IL6ST [74,75,76]. These differences in the complex formation mechanism are likely to contribute to distinct changes in the intracellular portion of gp130/IL6ST. Differential target response to signalling thresholds across cell types, as well as a range of modifications and many potential regulatory mechanisms also likely contribute to the plasticity and specificity in gp130/IL6ST-mediated signalling [70]. Additionally, crosstalk with other pathways through shared signalling components and factors in the cytoplasm adds to the modulation of a cytokine’s effect [77]. Authors have suggested that the tissue-specific effects of cytokines might be the result of signalling orchestration, where certain cell types can integrate the range of possibly opposing signals with interplaying mechanisms and factors for a balanced final response [78]. Further studies are needed to better elucidate the complex signalling machinery enabling functional specificity. In the meantime, this poses an interesting challenge in understanding how to tackle signalling of IL6 and other cytokines for therapeutic purposes, as effective agents would need to achieve a similar degree of specificity to enable targeting certain deleterious processes without compromising other essential activities (see Section 8).

## 5. The Role of the IL6-like Cytokine Family in BC

Among their broad range of pleiotropic functions, IL6-like cytokines are well-established as secretory factors contributing to many pro-carcinogenic changes, including disease progression or the development of treatment resistance, in a wide range of types of cancers [79,80,81,82,83]. This signalling axis is also involved in the regulation of homeostasis and other essential functions such as inflammation and immunity. In fact, the complex interaction between these functions and cancer has been thoroughly described [81,84,85]. For example, as pro-inflammatory signals regulated by these cytokines have been shown to the play a role in neoplastic aetiology and progression, and this could be exploited for therapeutic purposes. In the following paragraphs, we will focus on the role of these cytokines in BC, describing the activity and biomarker potential in this disease of different members of the IL6-like family.

### 5.1. IL6 in BC

#### 5.1.1. Signalling Role in BC

As the prototypical pro-tumourigenic member of its cytokine family, IL6 has been shown to exert a wide range of pro-cancer effects, including promoting tumour initiation and progression, survival, invasion, metastasis and chemo-resistance. The evidence on the role of IL6 in cancer has been reviewed extensively [19,86,87]. Here we will summarise the important roles of IL6, its downstream effectors and pathways in BC, as well as the role of IL6 as a marker in this disease.

STAT3, highly active in more than 50% of BCs [88,89], has been described as a key signalling orchestrator of many of the cancer-promoting effects exerted by IL6, but also IL11, LIF and OSM [70,79]. Evidence has also shown that STAT3 enables cross-talk of the JAK/STAT pathway with the other gp130/IL6ST-dependent pathways, contributing to cancer-promoting effects of the MAPK/MEK/ERK and PI3K/AKT signalling pathways, such as chemo-resistance and epithelial-mesenchymal transition (EMT) [90,91]. 

Numerous studies have assessed the effect of IL6 on proliferation in vitro [86,92], with diverging conclusions: while most evidence has suggested that recombinant IL6 can inhibit proliferation in ER-positive (ER+) cell line models [93,94,95,96,97,98], this has been contested by other studies and some have also shown a divergent motility-promoting effect [96,97]. Indeed, some mechanistic studies have shown that STAT3 can induce cell cycle progression and inhibit various apoptotic genes [19,72,99,100], while PI3K/AKT signalling inhibits p53, Chk-1, and transcription of tumour suppressors, and induces cyclin D1, myc, and mTOR transcription [101,102], with these changes contributing to proliferation and cell survival. Conversely, other studies have reported that IL6 might induce apoptosis and help inhibit proliferation in MCF-7 cells [93,95,103]. Interestingly, one study on ER+ cells in 3D culture found that IL6 could promote proliferation [104], while another on xenograft models found that IL6 increased expression of EMT-related genes through STAT3 [105]. Similarly, a study found that the blockade of gp130/IL6ST signalling had different effects in vitro compared to in vivo: inhibition led to higher proliferation in cell line models, but reduced malignancy in mice models [106]. This evidence from more complex models suggests that the effect of this cytokine on proliferation might be dependent on the TME. Another likely determining factor is receptor expression in the different models studied, but most in vitro studies have not considered the potential role of membrane-bound or soluble receptors [86].

The effect of IL6 on proliferation is only one of its many roles in BC. Studies have shown that IL6 can exert a pro-metastatic effect by modulating genes related to EMT, a process essential to the metastatic process, via STAT3 [107,108]. Transition from a stationary to a motile phenotype is also aided by the downregulation of E-cadherin expression, leading to loss of adhesion [97,105,107,109]. IL6 can also induce pro-angiogenic effects, inducing VEGF expression in tumour-associated endothelial cells through STAT3 and MAPK [110], while the IL6 inflammatory loop can activate mechanisms linked to drug resistance [111,112,113].

#### 5.1.2. Circulating IL6 Level as a Biomarker

Different sources of IL6 can induce a tumour-promoting effect in BC cells. Malignant cells are known to be a major source of IL6, with BC cells producing much higher levels of both the cytokines and its receptors than normal epithelial breast cells [114]. This endogenously-produced IL6 is used as a growth factor in an autocrine manner. In addition to production by cancer cells, tumour-associated macrophages (TAMs), helper T (Th) cells and tumour-associated fibroblasts have been shown to be primary sources of IL6 in the TME, suggesting these cell types enable paracrine IL6 signalling which can in turn contribute to oncogenesis and proliferation [104,115,116,117]. Interestingly, an IL6 activation signature revealed that pathway activity as measured in breast tumour samples correlated with circulating serum IL6 levels [118]. Both local autocrine and paracrine release are able to activate IL6 trans-signalling, thus contributing to the cytokine’s tumour-promoting effects [119,120,121,122]. In turn, these circulating cytokines could also enable endocrine IL6 signalling in distant lesions as the disease spreads.

Although pre-clinical studies have produced diverging evidence on the effects of IL6 and the utility of its in-tumour levels as a marker remains unclear [123,124,125,126], the role of circulating serum IL6 levels as a negative prognosticator in BC has been well established [79,92]. Indeed, compared to healthy women, serum IL6 levels are significantly elevated in BC patients and correlate with the stage of disease [127,128]. Higher levels were also observed in patients with widely dispersed metastatic BC compared to single metastatic disease, in recurrent compared with non-recurrent disease and in progressive compared to stable disease [128,129,130,131,132,133]. Elevated serum levels were also associated with worse prognosis and survival, as well as reduced response to chemo- or endocrine therapy [128,129,130,131]. Multivariate analysis confirmed that IL6 is an independent negative prognosticator in metastatic BC [130]. A meta-analysis of previous studies also found that high IL6 expression is associated with poor overall survival [134]. Although correlation with other clinical factors or tumour characteristics was not identified, the analysis was limited by the heterogeneity across studies in the different sources for IL6 detection (tumour vs. serum). In short, despite diverging reports on IL6’s role from in vitro studies, clinical evidence firmly supports that IL6 is involved in and a biomarker of BC development and progression. Despite the evidence on its prognostic and potentially predictive value of serum IL6 levels, prospective studies are needed before their assessment can be applied in BC detection and monitoring or to guide treatment selection.

Studies have reported changes in IL6 levels during treatment with taxane-based chemotherapy [135,136], but not with anthracycline-based chemotherapy [137,138] or endocrine therapy [139]. Some effort has gone into investigating a potential role of elevated cytokine level in the development of adverse events in patients receiving treatment for BC. Plasma IL6 levels were found to be associated with the development of fatigue in early-stage BC patients receiving radiotherapy [140,141], although other studies found no link between fatigue and IL6 levels in later stages [142,143]. Pre-treatment levels of IL6 were also higher in patients who went on to develop depression [144,145,146]. In long-term survival, plasma IL6 levels were associated with reduced cognitive function and poorer memory [147,148]. These findings suggest that anti-inflammatory therapies and, more specifically, agents inhibiting IL6 might help alleviate some of the side effects associated with anti-cancer treatment. 

Researchers have also studied whether the systemic IL6 level could be an indicator of BC risk in healthy women, since evidence of non-steroidal anti-inflammatory treatment leading to reduced risk of developing cancer suggested that circulating pro-inflammatory factors such as this cytokine might be linked to a higher predisposition to breast neoplasms [149]. No correlation between IL6 and breast cancer risk was found in two prospective studies in older populations, although the studies were limited by a low predictive power [150,151]. 

Circulating levels of sIL6R have been found to be elevated (in comparison to healthy individuals) in patients with different cancer types, including myeloma, leukaemia, bladder, prostate and hepatocellular cancer, with higher levels associated with tumour grade, volume or disease spread [152,153,154,155,156,157]. While significantly higher sIL6R levels have also been reported in patients with BC [158,159], it has been suggested that larger sample cohorts need to be assessed before conclusions can be drawn regarding the prognostic potential of sIL6R levels in this cancer type [27].

### 5.2. Other IL6-like Cytokines in BC

Other cytokines and associated receptors have also been associated with breast disease. As previously discussed, there is a certain degree of functional redundancy through the shared signal transducer and the 3 main downstream pathways described. For instance, some studies have shown that both IL6 and OSM are capable of inducing tumour-promoting effects through STAT3, while IL6, IL11, LIF and OSM can all promote invasion and metastasis through the JAK/STAT3 and PI3K/AKT pathways [79,160,161,162,163,164,165,166]. On the other hand, different cytokines can also trigger diverging responses through modulation of common mediators and signalling pathways. For instance, IL6 induces numerous cancer-promoting changes through STAT3, while IL27 or OSM can induce an opposing effect through the related signal transducer STAT1, also activated as part of the JAK/STAT pathway [167,168]. As is the case for IL6, other cytokines might exert a complex range of possibly opposite effects, with evidence of anti- and pro-tumour effects. In the next paragraphs we summarise some of the evidence to date on the role and potential clinical implications of some of the other IL6-like cytokines in BC.

#### 5.2.1. IL11

Having long been established as a haematopoietic growth factor, research in recent years has highlighted the potential pro-tumourigenic role of IL11 in epithelial cancers, with abundant evidence of its role in gastric cancer [169]. BC cell line studies have shown that, while only some models secreted IL11 [170], most expressed its specific receptor, IL11R [171,172]. In line with the typical signalling of cytokines in the IL6-like family, STAT3 is a central orchestrator of most of the known effects of IL11 in cancer, including promotion of cell growth and survival [173]. For example, studies on a triple-negative BC cell line model showed that blockade of IL11 led to increased response to chemotherapy [174]. The same researchers reported that higher IL11 levels were associated with poorer survival in BC patients treated with chemotherapy, suggesting this cytokine might play a similar role in vivo. 

Characterisation of a range of cancer cell lines, including a BC model, found that IL11 and IL11R expression was induced by the development of hypoxia [175]. STAT3 has also been shown to contribute to the effects of the hypoxia-inducible factor-1⍺ (HIF1⍺) transcription factor to promote angiogenesis, a key process in cancer progression and dissemination [176]. Animal studies using triple-negative human BC xenografts including IL11-overexpressing subclones have supported the role of IL11 in tumour growth, metastasis and angiogenesis [175,177].

The best-established role of IL11 in BC is in metastasis promotion. BC metastases to the bone are normally osteolytic, involving the activation of osteoclasts (bone-resorbing cells) that cause bone degradation and in turn enable tumour expansion. Recent animal studies have shown that IL11-driven activation of JAK/STAT signalling is an essential factor in promoting metastases-driven osteolysis [91]. Research has shown that different IL6-like cytokines are produced by osteoblasts (bone-forming cells) in physiological conditions and can be involved in normal bone remodelling [178,179,180]. Interestingly, IL11 is also expressed by bone marrow stromal cells and its secretion plays a central role in osteoclastogenesis [181]. The fact that IL11 is produced endogenously by stromal cells under physiological conditions and is essential to the formation and differentiation of bone-deforming cells (rather than just signalling for their activation like other related ligands) supports the notion that this cytokine plays a particularly important role in the bone microenvironment both during normal remodelling in healthy individuals and in the presence of colonising malignant cells [182]. Indeed, IL11 expression has been shown to correlate with risk of developing bone metastasis [183] and is higher in both tumour and serum samples from BC patients who presented these distant metastatic lesions [184]. 

Numerous other studies have investigated the clinical implications and potential prognostic value of IL11 expression. One study found that IL11 was elevated in tumour samples compared to matched normal breast tissue, regardless of subtypes and grade [172,185,186]. Another study showed higher levels of both IL11 and IL11R in clinical BC samples compared to normal breast tissue. Results also showed that IL11 was higher in tumours with node-positive status and poorer prognosis and that a higher level of the cytokine was linked to poorer survival [187]. Higher IL11 was also observed in patients who relapsed within 3–5 years compared to those who remained relapse-free [188]. 

Interestingly, a meta-analysis of 26 datasets from microarray studies found that higher IL11R expression was a positive prognosticator, associated with better survival in the lower-risk cohort of patients with negative node status [172]. Diverging from that earlier study, interrogation of multiple publicly-available datasets reported that IL11R was downregulated in most BC subtypes [172,189,190,191,192]. One notable exception is the mesenchymal stem cell-like subgroup of triple-negative BC. This subtype does express IL11R and presents an aggressive phenotype associated with poorer patient outcomes, suggesting the receptor might be a negative prognosticator [174]. The contrasting evidence on the role of IL11R as a biomarker in BC hints at the complex interaction between the receptor, its cytokine and other related factors, as well as at the fact that this signalling might vary across different BC subtypes.

#### 5.2.2. LIF

Evidence exists of a range of effects of LIF in BC. The role of LIF signalling on proliferation is unclear, with studies using the same ER+ cell line model reporting both growth-promoting [193,194] and anti-proliferative effects [171,195,196] of LIF. This suggests different factors might affect the role of LIF in promoting or inhibiting proliferation and further studies are needed to better characterise its function in BC. However, the proliferative effect of LIF has been reported in models expressing low LIFR, suggesting this activity is likely not dependent on this receptor. 

Although cell line studies have suggested that LIF can lead to increased migration and metastasis [164,165], its receptor LIFR has been shown to act as a breast tumour and metastasis suppressor through activation of the pro-dormancy activity of STAT3 [195,197,198,199,200,201]. In line with this, cell line models with low metastatic potential have been shown to express higher levels of LIFR and be responsive to LIF, whereas highly metastatic cells did not express the receptor and were unresponsive to the ligand [195]. Studies in ER+ cell line models have also shown that its knockdown leads to increased invasion, downregulation of dormancy genes and increased osteolytic bone destruction [195]. Overall, there is abundant evidence of a metastasis-supressing role of LIFR, suggesting a systemic pro-dormancy role in cancer cells disseminated to distant sites [195]. However, several cytokines (including LIF, OSM and CNTF) can recruit LIFR to initiate their signalling, so it remains unclear what ligands might drive this specific mechanism.

#### 5.2.3. OSM

Several studies have reported growth-inhibitory effects of OSM in BC cell line models [196,202,203,204,205], as well as in normal human mammary epithelial cells [206]. Conversely, cell line studies have also suggested that OSM might exert a pro-tumorigenic and pro-metastatic effect through induction of detachment, invasiveness, bone dissemination and EMT [160,207,208,209]. Studies in animal models showed that OSM knockdown reduced the formation of metastases, with findings suggesting that autocrine and paracrine signalling might be linked to metastasis to bone and lungs, respectively [210,211]. Data from clinical samples showed that patients with higher OSM levels had decreased survival, further supporting this role in metastasis. 

OSM can signal through both OSMR and LIFR [179,212], with evidence suggesting different downstream effects of OSM might be dependent on different signalling receptors [195,207]. Expression of OSMR has been shown to be associated with shorter recurrence-free and overall survival in BC patients [213], while evidence suggests a metastasis-suppressing role for LIFR. This supports the notion that OSM could have opposing effects depending on which receptor it binds to initiate its signalling, with OSMR potentially being involved in the cancer-promoting role of the ligand, while association with LIFR could be linked to its growth-inhibitory effect. Nevertheless, further work is needed before the complex signalling machinery and specific role of OSM in vivo can be fully elucidated.

### 5.3. IL6ST as a Biomarker in BC

As the cornerstone of all signalling by cytokines in the IL6-like family, the shared signal transducer gp130/IL6ST holds particular potential as both a therapeutic target and a candidate biomarker. We recently reviewed the extensive evidence of gp130/IL6ST as a promising predictor in BC [214]. In short, in recent years ten different independent studies based on the analysis of clinical BC samples have shown the value of gp130/IL6ST as a prognostic and/or predictive biomarker. Six studies reported that gp130/IL6ST serves as an independent marker and is, specifically, a positive prognostic marker in BC; its expression is significantly correlated with ER expression and better prognosis, and inversely correlated with adverse events such as invasion, metastasis and recurrence [214]. 

We also showed that IL6ST has been included in four different multifactor signatures (including the clinically-available EndoPredict assay), where gp130/IL6ST also served as a positive prognostic factor for ER+ BC [214]. These multigene signatures enable stratification of BC patients into prognostic groups with differing risks of recurrence and rates of response to different therapeutic strategies, so they could aid treatment selection. These findings suggest that inclusion of gp130/IL6ST, along with other molecular and clinicopathological factors, might provide further insight into the complex underlying biology of the disease and could, in turn, enable better patient stratification. As a result, these multifactor tools represent a promising avenue for the potential clinical translation of gp130/IL6ST’s value as a biomarker in BC.

## 6. IL6-like Cytokines and Oestrogen Signalling

Given the essential role that oestrogen and its signalling play in the majority of BCs, extensive efforts have gone into assessing their potential interaction with cytokine signalling. Research has shown a complex association between the oestrogen receptor and IL6 signalling. ER+ cells have been shown to be more responsive to IL6 than hormone-independent cells. Interestingly, an in vivo study showed that IL6 could drive engraftment of xenografts derived from an oestrogen-dependent BC cell line in the absence of hormonal supplementation and this could be blocked using an IL6 inhibitor [118]. Although earlier evidence suggested IL6 expression might correlate with ER [215], several more recent studies have shown that ER-negative (ER-) cultured models produce higher levels of the cytokine [216,217,218]; thus, ER- cells might be exposed to constant autocrine IL6 signalling, rendering them less sensitive to fluctuations in exogenous IL6 levels when compared to ER+ cells [219]. Research has also assessed a potential link between ER status and IL6R expression, with diverging results: cell line studies have suggested that ER+ cells release mainly sIL6R, whereas ER- cells express mIL6R [95]; however, a more recent study of serum samples showed that patients with ER+ tumours had lower levels of sIL6R compared to ER- patients [220]. 

IL6 can activate oestrogen-generating enzymes in both the tumour and adjacent tissues [126,221,222,223], acting as a key modulator of the conversion of estrone to estradiol [223]. Thus, IL6 can lead to an increase in local and circulating levels of oestrogen, as well as oestrogen sulfate, which can remain in circulation longer and acts as a hormone reservoir. Interestingly, research has shown that the IL6-like cytokines IL11 and OSM can stimulate aromatase expression via binding of STAT1/3, supporting the notion that cytokines secreted locally by cells in the TME contribute to upregulation of aromatase activity [224,225,226].

A study of primary BC-derived cultures showed that IL6 can cause direct transcriptional activation of ER [227]. This supports the notion that, even if lower levels of IL6 are produced endogenously in ER+ tumours, the cytokine still contributes to the advancement of ER-driven disease. Interestingly, cell line studies have reported that ER can trans-repress the expression of IL6, suggesting a potential negative feedback loop. This could explain why ER- cells, where this negative feedback loop is inactive, produce more IL6 [228]. In turn, this cytokine up-regulation has been suggested as a contributing factor to the greater invasive and metastatic potential of ER- breast cancer [92,106,228]. 

In vitro studies on endometrium and decidua cells have shown that oestrogen and ER signalling might play a role in determining the balance between soluble and membrane-bound forms of gp130/IL6ST [229]. This suggests that hormonal regulation could also modulate expression and release of the different isoforms in BC, thus altering cytokine signalling and its downstream effects on the disease. 

Researchers have also investigated the association between hormone receptor status and other cytokines and receptors in the IL6-like family. Studies have shown that expression of LIFR correlates with ER in clinical samples [230] and its level and function are also higher in ER+ cell line models [195]. Evidence has suggested that higher LIFR levels are associated with favourable biological features and better outcome and loss of LIFR favours bone metastasis [195,230]. In line with this, a recent study reported a metastasis-promoting mechanism in which ER is involved in down-regulation of LIFR expression, suggesting a potential negative feedback loop, similar to the one observed for IL6 [231].

Levels of OSM and its receptor OSMR have been shown to be inversely correlated with expression of ER and its target genes. Studies in ER+ cell lines also showed an antagonistic relation between ER and OSM and their associated signalling [213]. While the role of IL6-like cytokines in BC appears to be complex and the relation between ER status and the different cytokines in the family is heterogeneous, there is a common trend of evidence of ER-driven mechanisms of negative regulation of IL6, LIF and OSM signalling.

Lastly, the positive correlation between gp130/IL6ST expression and ER has been well established, as we recently reviewed [214] (also see Section 5.3). In short, evidence suggests that gp130/IL6ST might act as a robust surrogate marker of active oestrogen-related signalling. Importantly, its expression levels might provide an insight into the heterogenous underlying biology of ER+ BC, helping to stratify ER+ tumours into subsets that differ in their true level of hormone dependence and, consequently, their likelihood of response to endocrine therapy.

## 7. Polymorphisms in gp130/IL6ST-Dependent Signalling

Mutations leading to changes in the expression, activation or stability of molecules in the gp130/IL6ST signalling axis represent potential mechanisms for the development and progression of disease. Epigenetic alterations might also play an important role in aberrant activation of cytokine signalling in cancer. While the evidence to date has been reviewed extensively before [17,232], in this Section we will summarise the role polymorphisms in this protein family specifically as it pertains to BC. 

### 7.1. Polymorphisms in IL6-like Cytokines

While there is no evidence of naturally-occurring gain-of-function mutations in IL6, numerous studies have reported loss-of-function mutations that might alter its expression or downstream signalling and are linked to a range of pathologies [17]. In BC, mutations have mainly been described in the promoter region of the IL6 gene. The best characterised SNP, -174G/C (rs1800795), has been shown to cause IL6 overexpression at least in part by enabling recognition by other transcription factors [233,234]. However, the potential clinical implications of this mutation are unclear. Some studies found the mutant GG genotype was a negative prognosticator associated with reduced disease-free survival (DFS) in ER+ patients receiving chemotherapy [235] and with increased risk of metastasis irrespective of ER status [236]. In contrast, other studies found that a wild-type CC genotype was associated with a more aggressive phenotype and worse overall survival [237] or with lymphovascular invasion [238]. Another group also reported that C-carrying cancers had a higher risk of early events; this association was observed in ER- tumours, particularly after radiotherapy, but also irrespective of ER status for chemotherapy-treated cancers [239]. Overall, researchers have hypothesised that this polymorphism might alter the effect of the TME on IL6 expression: while wild-type tumours increase production of the cytokine in response of inflammatory stimuli such as radio- or chemotherapy or in ER- disease, mutant tumours might produce more IL6 regardless of systemic changes [239]. Other studies have suggested that hormonal status might influence how genetic variation affects the role of IL6 in BC [240]. Another polymorphism in the IL6 promoter is the SNP -597G>A (rs1800797), which has been linked to worse DFS and higher risk of early events [238,239,241]. Diplotypes including both -174G>C and -597G>A have also been associated with worse DFS (IL6-174G/C (rs1800795) [235]. 

Numerous studies have assessed the effect of IL6 polymorphisms on BC risk with inconsistent conclusions. For example, while some studies found that the -174G>C SNP led to an increased risk of disease [240,242], other studies and meta-analyses found no association between this or other polymorphisms in IL6 or IL6R and BC risk [234,243,244]. Interestingly, a large study evaluating 16 genes for interleukins and their receptors, which included over 100 SNPs, found that associations of polymorphisms (including some in IL6 and IL6R) with risk differed depending on ethnic background [245]; this suggests that heterogeneity in patient cohorts across different studies might contribute to the diverging results seen to date. Further work is needed to better elucidate the effect of IL6 SNPs on BC risk and progression, which most likely depends on several interacting factors. 

There is less knowledge on mutations in other IL6-like cytokines. SNPs in LIF, CNTF and OSM that lead to systemic cytokine deficiency have been linked to a broad range of pathologies [17]. However, there is little evidence of such aberrations contributing to cancer, although a recent study showed that polymorphisms in IL11 could affect susceptibility to gastric cancer [246]. 

### 7.2. Polymorphisms in Non-Signalling Receptors

Numerous polymorphisms in IL6R have been identified in humans, which largely alter the well-established role of IL6 in inflammation [247]. For example, the SNP p.D358A (rs2228145), affecting the site of cleavage by ADAM proteinases, leads to higher levels of sIL6R [248,249]. This has a systemic protective effect against inflammation that is translated into differential risks of inflammation-related conditions [249,250,251]. Another, rarer loss-of-function polymorphism in IL6R is linked with severe immune and inflammatory disorders [252].

Genotypic changes in IL6R can also affect BC, with a study finding that the rs11265608 SNP was associated with worse prognosis and reduced DFS [253]. Mutations in IL11R CNTFR have been linked to musculoskeletal alteration humans [254,255,256,257], but no aberrations in these receptors have been shown to affect BC. 

### 7.3. Polymorphisms in Signalling Receptors

As the cornerstone of signalling by IL6-like cytokines, aberrations in the signal transducer gp130/IL6ST have the potential to affect many downstream pathways and effects. Introduction of a knock-in gp130/IL6ST mutation was shown to cause hyperactivation of STAT3 through the disruption of a negative feedback mechanism, leading to the promotion of the development of adenocarcinomas [258]. In an important first finding in humans, a study of inflammatory hepatocellular adenomas reported small in-frame deletions in gp130/IL6ST in 60% of samples [259]. These gain-of-function somatic aberrations in the ligand-binding domain of gp130/IL6ST caused changes in intracellular distribution of the receptor and constitutive ligand-independent activation of STAT3 [259,260], exemplifying a novel mechanism for overactivation of pro-tumourigenic signals observed in many tumours. The inflammatory phenotype of these benign cancerous lesions evidences the role of IL6-like cytokines in both inflammation and cancer. 

Further work is needed to determine the role of these or other gp130/IL6ST aberrations in other epithelial cancers, including breast neoplasms. Interestingly, as binding of different cytokines involves specific residues in gp130/IL6ST, mutations might affect signalling by different ligands differently. For example, characterisation of a human mutation within a patient report showed that the N404Y mutation affected signal transduction of IL6, IL11, IL27 and OSM, but not LIF [261]. 

As for the other signalling receptors in the IL6-like cytokine family, there is no significant evidence of any activating mutations in OSMR or LIFR, although loss-of-function polymorphisms have been reported in patients with rare genetic disorders [262,263,264]. Polymorphisms in OSMR have been shown to be associated with increased risk and differential prognosis in different cancers [265,266,267], although no aberrations have been shown to play a role in BC.

### 7.4. Polymorphisms in Downstream Factors

Signalling can also be affected by polymorphisms in factors involved in the main pathways under gp130/IL6ST modulation, as has been extensively reviewed [232]. For example, both JAK2 mutations that lead to constitutive activation of JAK/STAT3 [268,269,270,271] and JAK2 fusion proteins [272,273,274,275] have been reported at high frequencies in patients with a range of pathologies. JAK polymorphisms have also been reported, albeit at a lower frequency, in solid tumours, including mutations in JAK1 and JAK3 in BC [271,276,277]. Activating mutations in these genes appear to function similarly, by blocking mechanisms of JAK activity autoinhibition [270,278]. Similar aberrations in the kinase TYK2, including activating mutations and gene fusions, have also been reported in myeloid disorders [279,280], but have not been observed in BC.

STAT3 mutations that lead to enhanced dimerisation and the constitutive activation of the JAK/STAT signalling cascade have been reported in leukemia [281,282] and lymphoma [283]. STAT3 polymorphisms have been detected in benign liver tumours [284], the same type of lesions shown to carry activating mutations in gp130/IL6ST [259], JAK1 and the kinase FRK [277], which also lead to constitutive activation of STAT3 [285]. 

Although mutations in STAT3 have not been reported in other solid tumours, there is extensive evidence of other aberrations causing downregulation of SOCS proteins or inhibition of SPH phosphatases that also lead to JAK/STAT dysregulation and STAT3 hyperactivity [286]. In BC, there is evidence of the expression and high signalling activity of STAT3 [89,287], which could be driven by aberrant JAK forms (reported in some clinical samples) or by other mutations affecting this signalling cascade. For instance, BC cell line models have been shown to carry activating mutations in Src kinases, which also modulate STAT3 and its effect on proliferation [288,289,290], and anti-STAT3 strategies has been shown to reduce tumour growth in animal studies [291]. 

These represent only some of the mechanisms that might affect the complex cytokine-driven signalling network in BC. While genotypic changes might vary between tumour types, it seems that alterations to the signalling cascade are a common feature across cancers and present a potential therapeutic strategy. Although this section has focused on polymorphisms in the main signalling axis JAK/STAT, aberrations with potential pro-tumourigenic effects also affect other pathways modulated by gp130/IL6ST, such as PI3K/AKT. For instance, in recent years much work has focused on the role and potential as therapeutic targets of PIK3CA genetic alterations, which are amongst the most common in BC [292,293].

## 8. Therapeutic Targeting of gp130/IL6ST Signalling

The involvement of IL6-like cytokines and their downstream signalling in many processes considered hallmarks of cancer has highlighted their potential as therapeutic targets. The pleiotropic role of these ligands means that blockade or inhibition of this signalling axis must be fine-tuned to prevent unwanted dysregulations. Despite this challenge, evidence shows that a high level of specificity can be achieved; for example, by recognising a critical residue in site III, the IL6 inhibitor olokizumab can hinder the interaction of gp130/IL6ST with the IL6-IL6R complex, but not other cytokines [294]. On the other hand, this pleiotropy also means that some drugs are already available that might be repurposed. A good example are anti-inflammatory agents that could also be used as anti-cancer treatments (see last paragraph in this section). 

Therapeutic approaches to date have included monoclonal antibodies for direct blockade of a ligand or receptor, recombinant cytokine regimes or small-molecule agents that interfere with downstream signalling. A variety of agents are at different stages of pre-clinical and clinical development and some are already in use. While emerging drugs targeting IL6-like cytokines and their signalling have been reviewed recently [19,79,87,88,295,296], in this section we will summarise the main therapeutic approaches and highlight those agents with current or emerging applications in BC.

IL6-mediated signalling might be blocked through direct inhibition of IL6 or IL6R using monoclonal antibodies. Promising pre-clinical evidence led to numerous clinical trials of the anti-IL6 antibody siltuximab to treat multiple myeloma or solid tumours, but these largely reported a lack of efficacy [79,295]. Although other novel anti-IL6 antibodies are being assessed for the treatment of immune disorders [297,298] or COVID19 (NCT04348500), further work is needed to evaluate their potential against BC.

The anti-IL6R antibody tocilizumab is already used to treat inflammation-related disorders and is currently in clinical trials to treat different cancer types. Following evidence from BC cell line models [113], a phase I trial is currently underway to assess use of tocilizumab in combination with HER2-targeted therapy to treat trastuzumab-resistant HER2+ metastatic BC (NCT03135171). Other anti-IL6R antibodies such as sarilumab and NI-1201 are currently also in development [299,300].

The characterisation of IL6-dependent signalling has suggested that specific targeting of its trans-signalling route might be a good strategy to block this cytokine’s pro-cancer effects without altering other important roles in homeostasis that normally rely on classic signalling [296]. Most drugs targeting IL6 or IL6R block both the classic and trans-signalling routes, although the emerging junctional epitope antibody VHH6, which binds the IL6-IL6R complex, has been shown to selectively inhibit trans-signalling [301]. The most common approach to achieve selective inhibition of trans-signalling has been the development of fusion proteins incorporating sgp130/sIL6ST, taking advantage of the natural antagonist role of the soluble form of the signal transducer. A prime example is olamkicept, a fusion product including the extracellular portion of the signal transducer (sgp130/sIL6ST) and the Fc portion (fragment crystallisable, a constant region of an immunoglobulin heavy chain) of a human IgG1 antibody [59]. This recombinant protein has been shown to exert a 10-fold greater inhibitory effect than sgp130/sIL6T and completely block IL6 trans-signalling both in vitro and in vivo [48,59,296,302]. Pre-clinical evidence has suggested the promise of olamkicept as an agent with potential to inhibit the role of IL6 in inflammation and cancer [79,302,303]. In theory, it could also block IL11 trans-signalling, although no evidence has been reported to date. 

Another strategy involves the direct targeting of gp130/IL6ST with small-molecule inhibitors such as SC144, which has shown promise in preclinical ovarian cancer models [304]. A small-molecule inhibitor named LMT-28, has also been shown to directly target gp130/IL6ST but only inhibits the effects of IL6 and not those of other cytokines in the family [305]. This supports the notion that, as with olokizumab’s targeting of IL6′s site III, the specific epitopes targeted by an inhibitor can determine which ligand-receptor complexes are blocked from binding the signal transducer. This might greatly impact the selectiveness of a given inhibitor’s effect on downstream signalling, so that finer blockade of the effects of a specific cytokine might be achieved [305]. Also in line with this, a recent study showed that targeted mutagenesis of different residues in CLC, which mediate interactions in the CNTF-CNTFR-LIFR-gp130/IL6ST signalling complex, can yield novel recombinant variants with distinct functions [306]. 

Of particular importance in the context of BC, the selective oestrogen receptor modulators (SERMs) raloxifene and bazedoxifene have been shown to inhibit the IL6-gp130/IL6ST interface [307]. Both agents are currently used to prevent and treat postmenopausal osteoporosis and raloxifene is also used to prevent BC in high-risk women [308,309]. Bazedoxifene has been shown to overcome hormone resistance in BC cells [310,311] and has a gp130/IL6ST-inhibiting effect in preclinical models of several cancer types [169,312,313,314,315]. Several BC clinical trials are currently underway: either in conjugation with oestrogens on benign proliferation or preinvasive breast lesions (NCT02729701, NCT02694809) or in combination with palbociclib to treat women with hormone receptor-positive breast tumours [316]. A recent study reported the development of novel bazedoxifene analogues designed to improve on the drug’s affinity for and targeting of gp130/IL6ST [317]. Results showed a lead analogue selectively inhibited IL6-dependent activation of JAK2 and STAT3 and suppressed tumour progression both in vitro and in vivo in xenograft lung cancer models. This evidence supports the promise of repurposing bazedoxifene and, now, its improved analogues for specific inhibition of gp130/IL6ST signalling in cancer treatment. This is particularly interesting for the management of BC, where this anti-oestrogen is already in clinical development and could potentially exert a double inhibitory effect.

While therapies targeting IL6-like cytokines such as IL11, OSM and CNTF are at different stages of development for the treatment of a range of diseases [17], only the IL11R-targeted agent BMTP-11 is being developed for its potential use in cancer treatment. There is preclinical evidence of its effect on several cancer types [318,319] and a prostate cancer clinical trial is currently underway [320].

Drugs might also target factors central to signalling downstream of the cytokine signalling complex, an approach with the potential to modulate the effect of most cytokines in the family. Pre-clinical studies have shown that JAK inhibition can inhibit growth of a wide range of cancer types, including breast [321,322]. Several ongoing studies are assessing the potential repurposing of ruxolitinib for cancer treatment [323], including an early phase clinical trial using this agent in combination with HER2-targeted therapy in BC (NCT02066532).

The key role of STAT3 in cytokine-dependent signalling also makes it an attractive therapeutic target but the development of effective inhibitors has proven difficult, due partly to the diffuse localisation of STAT3 in the cell and the high level of homology that complicates specific targeting of STAT3 alone and not other STAT proteins [324]. Despite some authors having labelled STAT3 an “undruggable” factor [79,325], several agents have now been developed to block its expression or function that have shown promise in pre-clinical cancer studies [79,88,324,326]. Some of these have now gone into clinical trials, with some promising preliminary results from a phase I study of OPB-51602, an inhibitor targeting the SH2 domain [327]. An interesting recent study has reported a proteolysis-targeted chimera (PROTAC) that enables potent and specific STAT3 degradation, with results showing complete tumour regression in mouse models of blood cancers [328]. This represents a novel strategy with potential for its application in other cancer types.

As for evidence in BC, the inhibitors G-quartet and S3I-201 have been shown to block STAT3′s ability to bind to DNA both in vitro and in vivo, with evidence of tumour regression in BC xenograft models [329,330]. Several drugs blocking STAT3 phosphorylation have also been shown to exert inhibitory effects in triple-negative cell line models [331,332,333,334]. However, further research is needed to better assess the therapeutic potential of any of these agents in BC. 

As previously mentioned, efforts to develop agents to block gp130/IL6ST in cancer will benefit from a better understanding of the signalling machinery, including the structure of cytokines and receptors and the specific residues involved in the recognition of protein partners and triggering of distinct downstream effects [324]. Better biomarkers are also needed to help guide selection of treatment plans that including targeted agents, either alone or in combination with other therapies. Indeed, evidence suggests that both combination treatments and the use of repurposed agents might be particular promising strategies [19]. For example, anti-IL6 therapies are already commonly used to manage side effects caused by the cytokine release syndrome in patients treated with immunotherapy, which can lead to over-activation of the gp130/IL6ST signalling and increased levels of IL6 [335,336,337,338,339,340]. In addition to alleviating these adverse effects and enabling better treatment adherence (see also Section 5.1.2), pre-clinical evidence has suggested that this combination treatments might also lead to a greater overall anticancer effect [79,340]. In line with this, research has also shown that, besides its pro-cancer effects, active JAK/STAT signalling also suppresses antitumour immune responses within the TME, suggesting that inhibition of this pathway might lead to a dual anticancer effect through activation of local immunity and also that combination with immunotherapy might enhance treatment response. On the other hand, clinical evidence has shown that IL6 inhibitors can also lead to immune-related side effects, such as increased infections in patients receiving tocilizumab [341]. 

## 9. Conclusions

Amongst their many functions, IL6-like cytokines play important roles in breast cancer. Both the prototypical member IL6 and the shared receptor gp130/IL6ST have been established as biomarkers with significant clinical potential in this disease. Other cytokines and receptors might also hold potential as predictors, as do specific polymorphisms in these molecules that continue to be investigated.

Extensive research has led to a better characterisation of the structure and complex interaction between these cytokines and receptors, as well as a more detailed understanding of their intricate downstream signalling. These advances have shed light on the potential for therapeutic targeting of this signalling axis in cancer. Evidence suggests that inhibition of trans-signalling might be a particularly promising strategy. Although the pleiotropic function of these cytokines means that a high level of specificity is needed to achieve effective targeting, numerous novel or repurposed agents are currently at different phases of assessment for their use as single or combination treatments. 

Further work is still needed to validate the role of some of these molecules as biomarkers and bring them closer to the clinic. Translation of this biomarker potential, which could help improve patient stratification and treatment selection, together with the potential application of the targeted agents currently under pre-clinical and clinical development, would represent a multi-pronged approach to exploit the central role of IL6-like cytokines in the management of cancer and, specifically, in breast neoplasms.

## Figures and Tables

**Table 1:** Members of the IL6-like cytokine family and their respective receptors.

Cytokine	Site I: Non-Signalling: Receptor	Site II: Signalling Receptor	Site III: Signalling Receptor
IL6	IL6R (IL6R⍺)	gp130/IL6ST	gp130/IL6ST
IL11	IL11R (IL11R⍺)	gp130/IL6ST	gp130/IL6ST
CLC	CNTFR (CNTFR⍺)	gp130/IL6ST	LIFR (LIFRβ)
CNTF	CNTFR (CNTFR⍺)	gp130/IL6ST	LIFR (LIFRβ)
CT1	-	gp130/IL6ST	LIFR (LIFRβ)
LIF	-	gp130/IL6ST	LIFR (LIFRβ)
NPN	-	gp130/IL6ST	-
OSM	-	gp130/IL6ST	LIFR (LIFRβ) or OSMR (OSMRβ)

CLCF1, cardiotrophin-like cytokine; CNTF, ciliary neurotrophic factor; CNTFR, CNTF receptor ⍺; CT1, cardiotrophin 1; IL6, interleukin-6; IL6R, IL6 receptor ⍺; IL11, interleukin-11; IL11R, IL11 receptor ⍺; IL27, interleukin-27; IL31, interleukin-31; gp130/IL6ST, glycoprotein 130, also known as IL6 signal transducer; LIF, leukemia inhibitory factor; LIFR, LIF receptor β; NPN, neuropoietin; OSM, oncostatin M; OSMR, OSM receptor β.

## Data Availability

Not applicable.
